# Rapid Quantification of the Latent Reservoir for HIV-1 Using a Viral Outgrowth Assay

**DOI:** 10.1371/journal.ppat.1003398

**Published:** 2013-05-30

**Authors:** Gregory M. Laird, Evelyn E. Eisele, S. Alireza Rabi, Jun Lai, Stanley Chioma, Joel N. Blankson, Janet D. Siliciano, Robert F. Siliciano

**Affiliations:** 1 Department of Medicine, Johns Hopkins University School of Medicine, Baltimore, Maryland, United States of America; 2 Department of Pharmacology and Molecular Sciences, Johns Hopkins University School of Medicine, Baltimore, Maryland, United States of America; 3 Howard Hughes Medical Institute, Baltimore, Maryland, United States of America; Emory University, United States of America

## Abstract

HIV-1 persists in infected individuals in a stable pool of resting CD4^+^ T cells as a latent but replication-competent provirus. This latent reservoir is the major barrier to the eradication of HIV-1. Clinical trials are currently underway investigating the effects of latency-disrupting compounds on the persistence of the latent reservoir in infected individuals. To accurately assess the effects of such compounds, accurate assays to measure the frequency of latently infected cells are essential. The development of a simpler assay for the latent reservoir has been identified as a major AIDS research priority. We report here the development and validation of a rapid viral outgrowth assay that quantifies the frequency of cells that can release replication-competent virus following cellular activation. This new assay utilizes bead and column-based purification of resting CD4^+^ T cells from the peripheral blood of HIV-1 infected patients rather than cell sorting to obtain comparable resting CD4^+^ T cell purity. This new assay also utilizes the MOLT-4/CCR5 cell line for viral expansion, producing statistically comparable measurements of the frequency of latent HIV-1 infection. Finally, this new assay employs a novel quantitative RT-PCR specific for polyadenylated HIV-1 RNA for virus detection, which we demonstrate is a more sensitive and cost-effective method to detect HIV-1 replication than expensive commercial ELISA detection methods. The reductions in both labor and cost make this assay suitable for quantifying the frequency of latently infected cells in clinical trials of HIV-1 eradication strategies.

## Author Summary

The eradication of HIV-1 from infected individuals is stymied by the persistence of the virus in a stable reservoir of latently infected CD4^+^ T cells. Latently infected cells can be found in all HIV-1 infected individuals at a very low frequency and allow the virus to persist despite antiretroviral therapy for the lifetime of an infected patient. Current efforts are focused on identifying small molecules or immune strategies to eliminate these latently infected cells. To assess the efficacy of these elimination strategies in HIV-1 infected patients, we must be able to measure the size of the latent reservoir. While an assay developed previously in our lab can measure the size of this latent reservoir, it is too laborious and costly to be utilized in large-scale HIV-1 eradication trials. We have developed a rapid assay to measure the size of the HIV-1 latent reservoir more amenable to these eradication trials.

## Introduction

Highly active antiretroviral therapy (HAART) has significantly reduced the morbidity and mortality associated with HIV-1 infection. However, while HAART can reduce plasma viral load to below the clinical limit of detection (50 copies HIV-1 RNA/mL) in adherent patients [1]–[3], this treatment is not curative. Even in individuals on prolonged suppressive HAART, HIV-1 persists as a latent but replication-competent provirus integrated in the genomes of a small percentage of resting memory CD4^+^ T cells [3]–[8]. These latently infected cells are extremely long lived as a consequence of the biology of memory T cells, with an estimated half-life of 44 months [9], [10]. The extreme stability of this HIV-1 reservoir precludes eradication with HAART alone and suggests that, without disruption of this reservoir, infected individuals must remain on HAART for the remainder of their lives [9], [10].

Recent studies have identified small molecules capable of reactivating HIV-1 gene expression [11]–[17]. While resting CD4^+^ T cells harboring a latent HIV-1 provirus are not susceptible to viral cytopathic effects or immune clearance, pharmacological reactivation of latent proviruses could lead to depletion of this latent reservoir. Recent *in vitro* work suggests that reactivation paired with a T cell vaccination strategy could be used to eradicate the latent reservoir of HIV-1 in resting CD4^+^ T cells [18]. Several clinical trials are investigating the ability of small molecule reactivators to perturb the latent state of the provirus and reduce the size of the latent reservoir [19], [20].

Assessing strategies to perturb or eliminate the latent reservoir requires assays that can accurately quantitate the size of the latent reservoir and that can be scaled for use in large clinical trials. The reservoir was originally identified using a viral outgrowth assay carried out on highly purified resting CD4^+^ T cells isolated from patient peripheral blood mononuclear cells (PBMC) [6], [7], [9], [21]. These resting cells do not actively produce virus without stimulation [4]. Limiting dilutions of the resting CD4^+^T cells are activated with the mitogen phytohemagglutinin (PHA) in the presence of irradiated allogeneic PBMC. This activation reverses latency and reinitiates the production of infectious HIV-1 from the subset of resting CD4^+^ T cells harboring replication-competent proviruses. The viruses that are produced are expanded in PHA-stimulated CD4^+^ lymphoblasts from uninfected donors, which are added to the culture at two time points. After two weeks, viral outgrowth is assessed by an ELISA assay for HIV-1 p24 antigen in the culture supernatant. The frequency of latent infection, expressed as infectious units per million (IUPM) resting CD4^+^ T cells, is determined using Poisson statistics. Typically, patients on long term HAART exhibit IUPM values between 0.1 and 1 [6], [7], [9].

While this viral outgrowth assay is widely recognized as the definitive assay for determining the minimum frequency of CD4^+^ T cells harboring replication-competent proviruses, it is time-consuming, labor-intensive, and expensive, requiring two weeks of cell culture and PBMC from at least three uninfected blood donors per assay. In its current form, this assay is not suitable for use in large clinical trials evaluating the efficacy of eradication strategies, and the identification of simpler assays for the latent reservoir has been identified as an AIDS research priority [22]. A recent study compared 11 different approaches for measuring persistent HIV-1 in patients on HAART [23]. Infected cell frequencies measured by PCR-based methods were generally at least two orders of magnitude higher than and poorly correlated with IUPM values. This likely reflects the presence of large numbers of defective proviruses that are detected by PCR-based assays. The results raise doubts about whether PCR-based assays can be used to assess the frequency of cells harboring replication-competent proviruses. Therefore we developed a rapid and simple viral outgrowth assay that can nevertheless detect and provide at least a minimal estimate of the frequency of cells that must be eliminated to cure HIV-1 infection.

## Results

### Patient characteristics

A rapid and simple viral outgrowth assay was developed and evaluated using samples from 20 patients with HIV-1 infection. The baseline characteristics of the patient cohort enrolled in this study are summarized in Table 1. Seventeen patients were recruited on the basis of prolonged suppression of viremia to <50 copies of HIV-1 RNA/mL on HAART, with a duration of suppression from 12 to 156 months. We intentionally enrolled three additional patients who were viremic at the time of blood draw with viral loads of 5,392, 452,059, and 31,238 copies/mL. Of the three viremic patients, one patient had not yet started HAART and two were known to be non-adherent. The average age of the cohort (± SD) was 50.8.±10.8 years, and the cohort was overwhelmingly comprised of black males. The CD4 nadir of the cohort ranged from 6 to 755 cells/µL, and 8 patients had a history of an AIDS diagnosis.

**Table 1:** Characteristics of study patients.

				Duration of HIV-1 Infection (months)	CD4 Cell Count (cells/µL)		Viral Load at Enrollment (copies/mL)	Time on HAART§ (months)	
Pt. ID	Age (years)	Sex	Race†		Nadir	Current			HAART Regimen‡
S1	57	M	W	288	157	806	<50	144	TDF, FTC, EFV, DRV/r
S2	54	M	W	132	321	554	<50	120	TDF, FTC, EFV
S3	27	M	W	24	511	638	<50	24	DRV/r, RAL
S4	62	F	B	240	6	721	<50	156	TDF, FTC, RAL/r
S5	77	M	B	72	182	357	<50	48	TDF, 3TC, RAL
S6	55	M	B	144	180	627	<50	96	TDF, FTC, ATV/r
S7	44	M	B	168	9	711	<50	156	TDF, FTC, EFV
S8	53	M	B	180	95	622	<50	120	TDF, FTC, ATV/r
S9	48	M	B	72	276	458	<50	144	TDF, FTC, EFV
S10	52	F	B	132	384	626	<50	144	TDF, FTC, EFV
S11	46	M	W	240	483	696	<50	48	TDF, FTC, DRV/r, RAL, MVC
S12	64	M	B	228	100	459	<50	84	DRV/r, RAL
S13	47	M	B	36	755	755	<50	12	TDF, FTC, DRV/r
S14	41	F	B	108	255	949	<50	72	TDF, FTC, FPV/r
S15	48	M	W	264	289	721	<50	84	TDF, FTC, EFV, ATV/r
S16	50	F	W	168	243	563	<50	15	TDF, FTC, EFV
S17	36	M	B	60	422	513	<50	40	TDF, FTC, NVP
V1	52	M	H	180	N/A	354	5,392	Viremic	Treatment Naïve
V2	60	M	B	168	210	223	452,059	Viremic	Non-adherent
V3	42	M	B	168	43	468	31,238	Viremic	Non-adherent

† Race abbreviations: W, white, non-Hispanic; H, Hispanic; B, black.

§ Time of documented continuous suppression of plasma viremia to <50 copies/mL on HAART.

‡ Drug abbreviations: DRV/r, darunavir boosted with ritonavir; FTC, emtricitabine; TDF, tenofovir disoproxil fumarate; EFV, efavirenz; RAL, raltegravir; RAL/r, raltegravir boosted with ritonavir; ATV/r, atazanavir boosted with ritonavir; 3TC, lamivudine; MVC, maraviroc; FPV/r, fosamprenavir boosted with ritonavir; NVP, nevirapine.

### Highly purified resting CD4^+^ T cells can be obtained using a two-step bead depletion procedure

**Figure 1:**
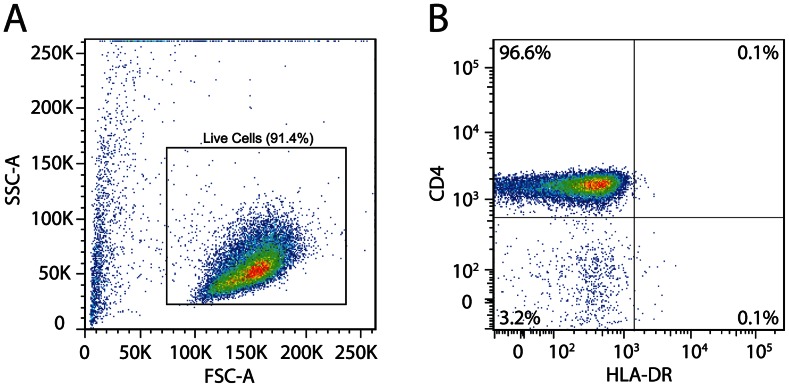
Two-step bead depletion procedure yields highly purified resting CD4^+^ T cells from HIV-1 infected patients. A two-step negative selection strategy to purify resting CD4^+^ T cells from patient PBMC. (**A**) Representative FSC/SSC plot indicating live cell population after the two-step bead depletion procedure. (**B**) Representative dot plot indicating purity of resting CD4^+^ T cells. Purified cells were stained with antibodies to CD4 and HLA-DR.

The original viral outgrowth assay used to define the latent reservoir was performed on resting CD4^+^ T cells purified from PBMC in a multistep process that included fluorescence activated cell sorting (FACS) and required a BSL-3 sorting facility. Resting CD4^+^ T cells were differentiated from activated CD4^+^ T cells by the absence of cell surface markers CD69, CD25, and HLA-DR. To simplify the purification of resting CD4^+^ T cells, we devised a two-step bead depletion purification procedure. Initially, CD4^+^ T cells were purified from PBMC by negative selection as described in Materials and Methods. Subsequent bead depletion of cells expressing CD69, CD25, or HLA-DR yielded a highly purified, unmanipulated population of resting CD4^+^ T cells. The purity of these cells was routinely assessed by staining with antibodies to CD4 and HLA-DR. Representative examples are shown in Figure 1 and Figure S1. Typical purities were 96–97% with less than 0.1% contamination with activated (HLA-DR^+^) CD4^+^ T cells. As is discussed below, IUPM values obtained with these purified resting CD4^+^ T cells were in the same range as observed with sorted cells.

### The use of MOLT-4/CCR5 cells for virus expansion gives statistically comparable results at 14 days

**Figure 2:**
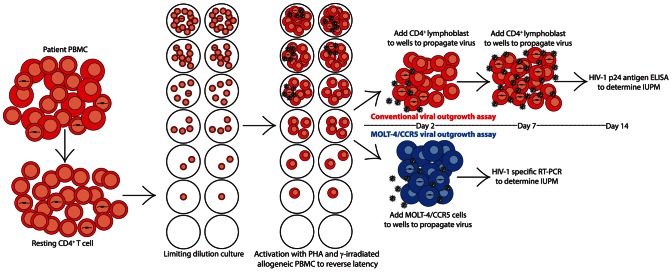
The standard and the MOLT-4/CCR5 viral outgrowth assays. The frequency of HIV-1 latent infection of resting CD4^+^ T cells can be measured using a viral outgrowth assay. PBMC are collected from HIV-1 infected individuals and resting CD4^+^ T cells (CD25^−^, CD69^−^, HLA-DR^−^) are purified. Resting T cells are plated in 5-fold serial dilutions in duplicate, such that the input number of patient cells ranges from 1,000,000 to 320 cells per well. To reverse latency in the cells that harbor a replication-competent HIV-1 provirus, patient cells are activated with PHA and a 10-fold excess of irradiated PBMC from healthy donors. The next day, target cells for HIV-1 infection are added to allow outgrowth of replication-competent HIV-1 released from infected cells in which latency has been reversed. In the standard viral outgrowth assay, CD4^+^ lymphoblasts from healthy donors are added on days 2 and 7 of the assay. In the MOLT-4/CCR5 viral outgrowth assay, MOLT-4/CCR5 cells are added on day 2 only. For the standard assay, HIV-1 p24 antigen ELISA is used to identify wells positive for HIV-1 outgrowth at 14 days. For the MOLT-4/CCR5 assay, RT-PCR is used to identify wells positive for outgrowth at 7 days. The frequency of latently infected cells can be determined using limiting dilution statistics based on the input number of patient cells in the wells positive for outgrowth. This frequency is reported in infectious units per million (IUPM).

**Figure 3:**
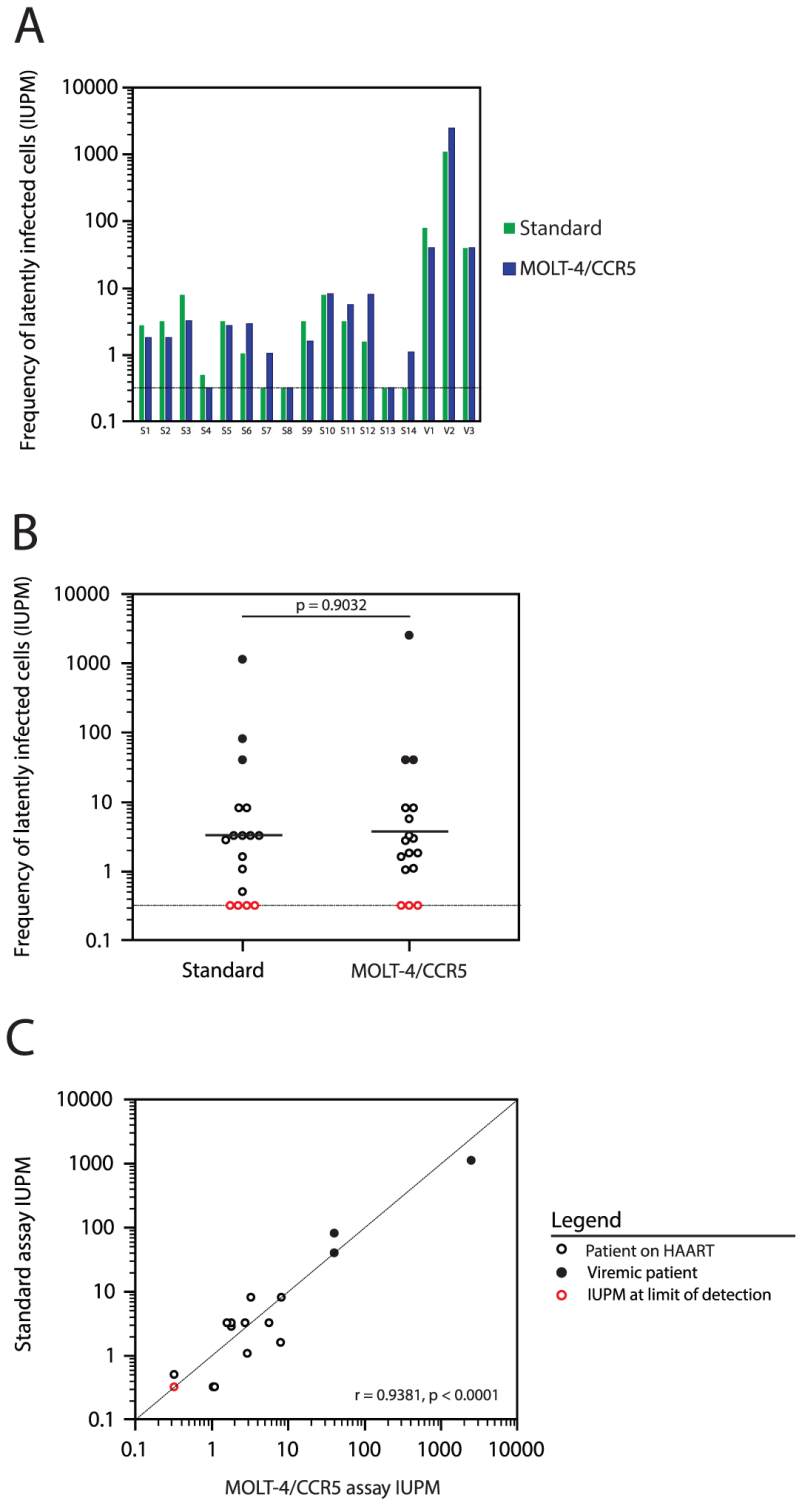
The standard and the MOLT-4/CCR5 viral outgrowth assays yield comparable frequencies of latent infection. (**A**) The frequency of latently infected resting CD4^+^ T cells was measured in 3 viremic patients and 14 HAART-suppressed patients using both the standard and the MOLT-4/CCR5 viral outgrowth assays, with HIV-1 p24 antigen ELISA used as the endpoint assay of viral outgrowth at day 14. (**B**) Statistical comparison of the IUPM values measured using the standard viral outgrowth assay and the MOLT-4/CCR5 viral outgrowth assay by Wilcoxon rank sum test. (**C**) The correlation of IUPM values measured using the standard viral outgrowth assay and the MOLT-4/CCR5 viral outgrowth assay (Pearson's correlation coefficient, r).

The standard viral outgrowth assay requires a minimum of 3 separate blood samples from healthy donors in addition to a blood sample from the test patient (Figure 2). CD4^+^ T lymphoblasts from two of these samples are added to cultures at days 2 and 7 to expand virus released from patient cells in which latency has been reversed by T cell activation. These donor lymphoblasts are prepared by PHA stimulation of donor PBMC for 2 days followed by depletion of CD8^+^ T cells. Eliminating the need for donor lymphoblasts in virus expansion would significantly simplify the assay. We hypothesized that a single addition of a cell line expressing high levels of CD4 and the co-receptors CCR5 and CXCR4 would allow for efficient expansion of viruses released from latently infected cells. We chose the MOLT-4/CCR5 cell line [24]. This cell line was derived from MOLT-4 cells [25], which express high levels of CD4 and CXCR4, and has been engineered to stably express CCR5. It is thus capable of supporting replication of both X4-tropic and R5-tropic variants of HIV-1. To verify that the MOLT-4/CCR5 cells are an acceptable alternative for donor-derived CD4^+^ T lymphoblasts, we compared IUPM values obtained using the standard viral outgrowth assay with donor derived lymphoblasts to IUPM values obtained using a simplified assay in which a single addition of MOLT-4/CCR5 cells was used in place of donor lymphoblasts. All 3 viremic patients (V1, V2, V3) and 14 of the 17 patients on suppressive HAART (S1–S14) were included in this comparison. Resting CD4^+^ T cells obtained from each patient using the bead depletion method described above were split and tested using the standard assay and the simplified MOLT-4/CCR5 assay as outlined in Figure 2. The p24 ELISA at day 14 was used as an assay endpoint. Replication-competent HIV-1 was isolated from purified resting CD4^+^ T lymphocytes in all 3 of the viremic patients and 10 of the 14 patients on HAART by both assays (Figure 3A, Table S1). In 3 of the 14 patients on HAART, replication-competent HIV-1 was isolated in only one of the two viral outgrowth assays (Figure 3A, Table S1, Patients S4, S7, and S14). No replication-competent HIV-1 was recovered from purified resting CD4^+^ T lymphocytes by either viral outgrowth assay in 2 of the 14 patients on HAART (Figure 3A, Table S1, Patients S8 and S13). These results are expected because splitting the sample reduces the input number of resting CD4^+^ T cells. The frequency of latently infected resting CD4^+^ T lymphocytes was markedly higher in the viremic patients compared to patients on HAART (Figure 3A, Table S1), consistent with our previous results [10].

No significant difference was observed between the frequency of latently infected resting CD4^+^ T lymphocytes measured in the standard viral outgrowth assay versus the MOLT-4/CCR5 viral outgrowth assay (Figure 3B, Wilcoxon rank sum test, p = 0.9032). Furthermore, the frequency of latently infected cells as measured by the MOLT-4/CCR5 viral outgrowth assay correlates highly with that of the standard viral outgrowth assay (Figure 3C, Pearson's correlation coefficient, r = 0.9381, p<0.0001). When only patients on suppressive HAART were considered, the correlation remained highly significant (r = 0.7602, p = 0.0016).

### HIV-1 specific RT-PCR assay detects HIV-1 outgrowth from the latent reservoir earlier than p24 antigen ELISA

**Figure 4:**
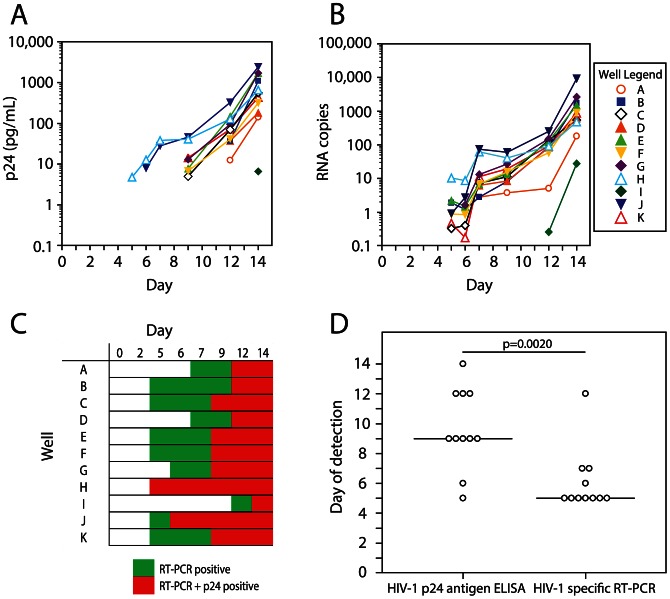
Kinetics of HIV-1 outgrowth from latently infected CD4^+^ T cells measured by HIV-1 p24 antigen ELISA and HIV-1 specific RT-PCR. Resting CD4^+^ T cells were isolated from HAART patient S15, whose latent reservoir was previously measured to be 3.25 IUPM. Twenty-nine replicate wells were plated in which 200,000 resting cells were activated with PHA and irradiated PBMC from a healthy donor and subsequently cultured with MOLT-4/CCR5 cells. Outgrowth of reactivated HIV-1 was measured in positive wells over 14 days using both (**A**) HIV-1 p24 antigen ELISA and (**B**) HIV-1 specific RT-PCR. (**C**) The difference between the day on which a particular well becomes positive by RT-PCR versus p24 ELISA. (**D**) The day of detection of HIV-1 outgrowth from the latent reservoir is shown for HIV-1 p24 antigen ELISA and HIV-1 specific RT-PCR.

To shorten the time required to measure latently infected cells by the viral outgrowth assay, we explored the use of RT-PCR as an alternative to the p24 ELISA to detect virus production. Twenty-nine replicate wells were set up with 200,000 patient resting CD4^+^ T cells/well from a patient on suppressive HAART (S15). The cells were activated with PHA and irradiated feeders and then cultured with MOLT-4/CCR5 cells over 14 days using the protocol developed for the viral outgrowth assay. Culture supernatants were assayed for released virus at multiple time points during the 14 day culture using both the p24 antigen ELISA and a novel RT-PCR assay. This assay detects polyadenylated HIV RNAs without interference from proviral or plasmid DNA, and when applied to virion-containing supernatants, detects mainly genomic viral RNA, allowing accurate quantitation of virus release [26]. For both assays, positive wells showed an exponential increase in the amount of virus in the supernatant (Figures 4A and 4B). Among the wells positive for outgrowth, there was complete concordance between HIV-1 p24 antigen ELISA and HIV-1 specific RT-PCR at 14 days. All of the wells that were positive for outgrowth by RT-PCR eventually tested positive by p24 ELISA (Figure 4C). Under conditions where a majority of the positive wells are predicted to contain a single latently infected cell, HIV-1 specific RT-PCR detected viral outgrowth significantly earlier than HIV-1 p24 antigen ELISA (Figure 4D, Wilcoxon rank sum test, p = 0.0020), with the average days (± S.D.) of detection being 6.1±2.1 and 9.6±2.7 days, respectively. These results indicate that HIV-1 specific RT-PCR accurately detects viral outgrowth in a shorter time frame.

### Accurate measurement of the frequency of latently infected cells can be obtained in 7 days using RT-PCR to detect outgrowth in the MOLT-4/CCR5 viral outgrowth assay

**Figure 5:**
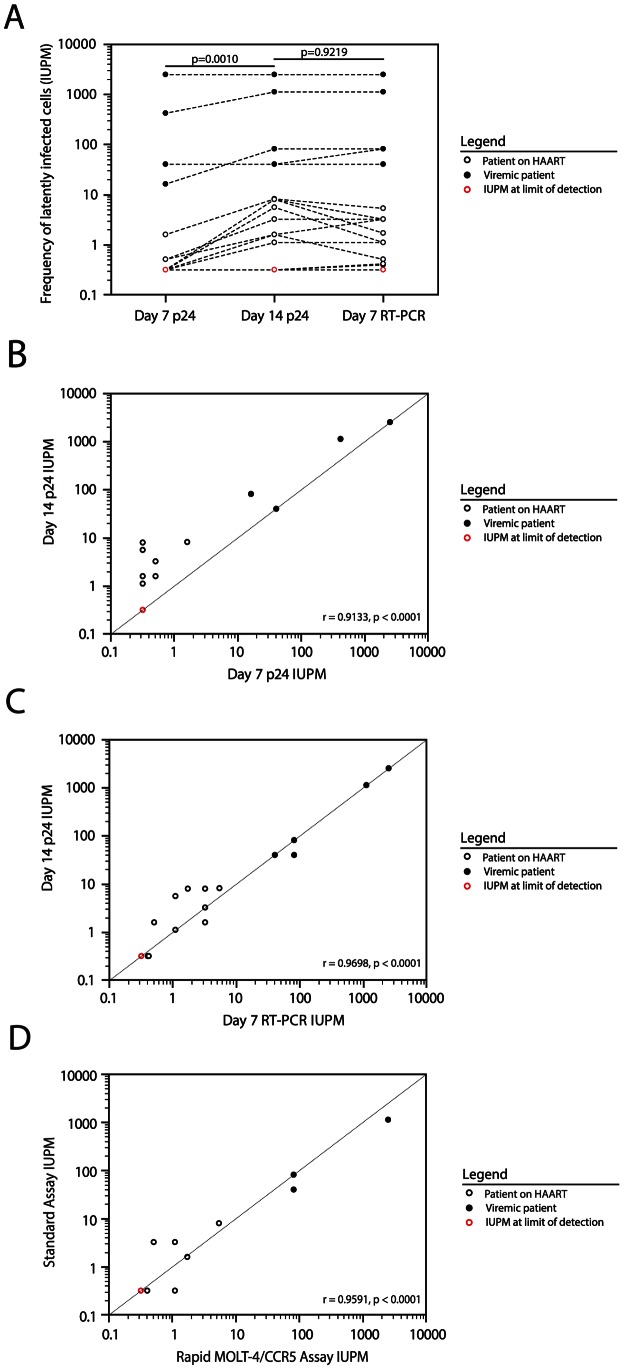
Accurate measurement of IUPM at day 7 using HIV-1 specific RT-PCR. (**A**) Using the rapid MOLT-4/CCR5 viral outgrowth assay, the frequency of latently infected cells was measured for HAART patients S1–S14 and viremic patients V1–V3 at day 7 with the HIV-1 specific RT-PCR assay and at both days 7 and 14 with HIV-1 p24 antigen ELISA. Statistical significance of the differences in IUPM values was assessed by Wilcoxon rank sum test. (**B**) Correlation of the IUPM measured at day 7 using HIV-1 p24 antigen ELISA with the IUPM measured at day 14 using HIV-1 p24 antigen ELISA (Pearson's correlation coefficient, r). (**C**) Correlation of the IUPM measured at day 7 using HIV-1 specific RT-PCR with the IUPM measured at day 14 using HIV-1 p24 antigen ELISA (Pearson's correlation coefficient, r). (**D**) Correlation of the IUPM measured at day 7 using the rapid MOLT-4/CCR5 outgrowth assay with the IUPM measured at day 14 using the standard outgrowth assay (Pearson's correlation coefficient, r).

Given that HIV-1 specific RT-PCR accurately detected HIV-1 outgrowth from the latent reservoir significantly earlier than the HIV-1 p24 antigen ELISA, we sought to determine whether HIV-1 specific RT-PCR could be used to detect positive wells more rapidly in the MOLT-4/CCR5 viral outgrowth assay. As shown in Figure 4B, nearly all wells that eventually became positive by p24 ELISA were positive by RT-PCR on day 7. Therefore, supernatants from viral outgrowth cultures from patients S8–S14 and V1–V3 were tested at day 7 using the HIV-1 specific RT-PCR assay. For comparison, an HIV-1 p24 antigen ELISA was also performed on culture supernatants from these viral outgrowth cultures on day 7. The frequencies of latently infected cells obtained using both assays on day 7 of the viral outgrowth assay were compared to the frequencies determined on day 14 using the HIV-1 p24 antigen ELISA (Figure 5A). The frequencies determined with HIV-1 p24 antigen ELISA on day 7 were significantly lower than the frequencies determined with the same assay on day 14 (Figure 5A, Wilcoxon rank sum test, p = 0.0010). However, the frequencies determined with HIV-1 specific RT-PCR on day 7 and with HIV-1 p24 antigen ELISA on day 14 were not significantly different (Figure 5A, Wilcoxon rank sum test, p = 0.9219). These results suggest that the use of a sensitive assay for free virus on day 7 on the culture may effectively substitute for an ELISA assay on day 14.

We next sought to evaluate the agreement between the day 7 and day 14 endpoint assays. A total of 74 wells from patients S8–S14 and V1–V3 were positive for HIV-1 outgrowth by either HIV-1 specific RT-PCR or HIV-1 p24 antigen ELISA. Outgrowth was detectable on day 7 using HIV-1 p24 antigen ELISA in only 60% of the positive wells. However, outgrowth was detectable in 81% of the positive wells on day 7 using HIV-1 specific RT-PCR. The correlation of the frequency of latently infected resting CD4^+^ T cells calculated at day 7 and day 14 was evaluated using Pearson's correlation coefficient. The correlation between the frequency of latent infection calculated at day 14 using HIV-1 p24 antigen ELISA and the frequency determined on day 7 was markedly higher when the HIV-1 specific RT-PCR assay was used rather than the HIV-1 p24 antigen ELISA (Figure 5C versus Figure 5B, r = 0.9698 versus r = 0.9133). When only patients on suppressive HAART were considered, the frequency determined at day 7 using the RT-PCR assay was positively correlated with the frequency determined at day 14 by HIV-1 p24 antigen ELISA (r = 0.8516, p = 0.0001) while the frequency determined at day 7 using the HIV-1 p24 antigen ELISA no longer correlated with the frequency determined at day 14 by ELISA (r = 0.4448, p = 0.1110). Two additional patients on suppressive HAART (S16, S17) were included in a final comparison of the rapid MOLT-4/CCR5 viral outgrowth assay and the standard viral outgrowth assay. A statistically significant positive correlation was seen when the frequency of latently infected resting CD4^+^ T cells determined using the rapid MOLT-4/CCR5 viral outgrowth assay was compared to the frequency determined using the standard viral outgrowth assay (Figure 5D, r = 0.9591, p<0.0001). When only patients on suppressive HAART were considered, a statistically significant positive correlation was still observed (r = 0.7522, p = 0.0194).

## Discussion

Latent HIV-1 infection of resting CD4^+^ T cells remains the major barrier to HIV-1 eradication. A number of small molecules have been identified that are capable of reactivating transcription of otherwise silent HIV-1 proviruses [11]–[15], [17]. Some of these compounds have already entered clinical trials [19], and drug discovery efforts to find additional compounds that can perturb or eliminate latent HIV-1 continue. Concurrently, immunological approaches are being investigated and have shown promise [18]. However, a key hurdle facing HIV-1 eradication efforts has, until recently, been largely ignored: the development of a reliable and simple assay to measure the size of the HIV-1 latent reservoir. Such an assay is absolutely required for evaluating the effectiveness of an eradication strategy. PCR based assays are being used to quantify proviruses in T cell subsets and the level of residual viremia in HIV-1 infected patients [19], [27]–[31]. A recent study has compared results of various PCR based assays with those obtained with the viral outgrowth assay using a set of samples from two well characterized cohorts of patients on HAART [23]. Because current PCR assays detect both replication-competent and defective proviruses, the correlation between infected cell frequencies measured by PCR and viral outgrowth was not strong, with the exception of an assay measuring integrated HIV-1 DNA in PBMC [23]. The measurement of integrated HIV-1 DNA by *Alu* PCR [30] is of particular interest because the stable reservoir for HIV-1 consists of resting CD4^+^ T cells harboring integrated HIV-1 DNA [4], [5]. It is likely that this and other PCR based assays will play an important complementary role to viral outgrowth assays. Prior to the present study, the standard viral outgrowth assay was the only assay available to directly quantify the frequency of resting CD4+ T cells harboring latent but replication-competent viral genomes.

The development of a rapid isolation procedure to obtain unperturbed resting CD4^+^ T cells was an essential first step towards creating a viral outgrowth assay suitable for widespread use in eradication studies and clinical trials. The latent reservoir was originally defined using viral outgrowth assays performed on highly purified populations of resting CD4^+^ T cells obtained through a combination of magnetic bead depletion and cell sorting. Thus, the assay required a BSL-3 cell sorting facility. While feasible for small-scale studies, this approach cannot be utilized for large-scale studies in which numerous measurements of the size of the latent reservoir must be taken across many patients. As we have demonstrated here, our isolation procedure yields a highly purified resting CD4^+^ T cell population. Moreover, the frequencies of latently infected cells measured in these populations are very similar to those obtained with the sorting method [4]–[6].

The standard viral outgrowth assay relies on a minimum of 3 separate blood samples from healthy donors. PBMC from two of the subsequent donations are added to the cultures to propagate the HIV-1 released following the reversal of latency. These healthy donor cells may also provide subsequent allogeneic stimulation to the patient cells in culture. We sought to replace these healthy donor cells with a cell line that supports infection by both X4-tropic and R5-tropic HIV-1. Furthermore, we believed that a cell line would provide greater uniformity to the viral outgrowth assay, since every viral isolate growing out of a patient's latent reservoir would propagate in identical culture conditions. As we have clearly demonstrated, the MOLT-4/CCR5 cell line performs robustly in place of mitogen stimulated CD8-depleted healthy donor PBMC in the viral outgrowth assay. This modification significantly simplifies the assay and allows the assay to be more easily scaled for large studies or clinical trials. Other cell lines that support replication of both X4 and R5 isolates could potentially be used as target cells in this assay. Of note, our rapid assay in its current form still requires a single blood donation from a healthy donor for the generation of irradiated PBMC used in the initial mitogen stimulation. It is possible that the replacement of mitogen stimulation with co-stimulation via anti-CD3 and anti-CD28 monoclonal antibodies could alleviate the need for any healthy blood donors.

Interestingly, the success of MOLT-4/CCR5 cells in propagating reactivated HIV-1 suggests that the allogeneic stimulation provided by the healthy donor CD4^+^ T lymphoblasts was not required. As MOLT-4/CCR5 cells do not express MHC class II [32], [33], no allogeneic stimulation of patient CD4^+^ T cells should occur. The lack of allogeneic stimulation was noted in the early characterization of the MOLT-4 cell line [34]–[36]. Thus, we can infer from our study that the initial mitogen stimulation alone is generally sufficient for reactivation of latent HIV-1 and viral outgrowth. However, we cannot conclude that this initial mitogen stimulation is sufficient for reactivation of all latent proviruses. Studies are ongoing to determine whether any replication-competent proviruses remain non-induced after a single round of mitogen stimulation. If this is the case, any measurement of the size of the latent reservoir that relies upon reactivation of latent proviruses through a single round of T cell stimulation may in fact be underestimating the size of the reservoir. The strategic use of both culture and PCR based assays may allow us to bracket the true size of the latent reservoir.

The standard viral outgrowth assay requires 14 days to complete. This 14 day period includes two additions of CD4+ T lymphoblasts from healthy donor PBMC as well as multiple media changes. The length of time required for the standard viral outgrowth assay is a function of the endpoint assay used to measure viral outgrowth: the HIV-1 p24 antigen ELISA. With the goal of reducing the length time required for detecting viral outgrowth, we adapted a recently developed HIV-1 specific RT-PCR assay for use as an endpoint assay. On average, viral outgrowth from the latent reservoir under conditions resembling the MOLT-4/CCR5 viral outgrowth assay was detectable by the HIV-1 specific RT-PCR assay after 6.1±2.1 days (± S.D) versus 9.6±2.7 days (± S.D) for HIV-1 p24 antigen ELISA. This result indicated that utilizing a more sensitive endpoint assay for viral outgrowth could indeed reduce the length of time required to complete the MOLT-4/CCR5 viral outgrowth assay. The data presented here demonstrate that no significant difference exists between the frequency of latent infection of resting CD4^+^ T cells as measured at day 7 using HIV-1 specific RT-PCR and at day 14 as measured by HIV-1 p24 antigen ELISA. The utilization of an RT-PCR based measurement for HIV-1 outgrowth not only allows more sensitive detection of viral replication, but will allow more high-throughput measurement of HIV-1 replication. Of note, extremely high sequence conservation has been observed at the primer and probe binding sites, especially amongst subtype B isolates [26]. The degree of conservation is actually higher than is observed in the regions of *gag* that are amplified in many other PCR assays [26]. It remains possible that in rare patients, sequence variation in these conserved regions could interfere with PCR detection. In these rare cases, negative results in the PCR assay may be due to primer mismatch rather than low frequency of latent infection.

Given the greater sensitivity of the HIV-1 specific RT-PCR assay, it is possible that small amounts of replication-defective virus released after mitogen stimulation of resting CD4^+^ T cells might be detected, resulting in a false positive readout of viral outgrowth. Our data suggest that the release of replication-defective virus is not widely detected by the RT-PCR assay at day 7. In rare cases (patient S13), we observed weak positive signals by RT-PCR at day 7 in wells that remained negative by p24 ELISA at day 14. These data could represent viruses that have low fitness and a slower replication rate than needed to expand to beyond the limit of detection for HIV-1 p24 antigen ELISA on day 14. It is possible that such viruses could be detected by HIV-1 p24 antigen ELISA with a longer culture period. As shown in Figure 4C, only 11 out of 29 wells containing an input of 200,000 resting CD4^+^ T cells from patient S15 were positive for viral outgrowth. The frequency of latent HIV-1 infection measured in the resting CD4^+^ T cells of patient S15 (3.25 IUPM) suggests that each of the 29 replicate wells likely contained dozens of integrated, defective proviruses. However, only 11 wells were positive for when tested by RT-PCR. Furthermore, of the wells that were positive for viral outgrowth, detection by HIV-1 specific RT-PCR was not possible until day 6.1±2.1 days (± S.D), and these wells remained positive for the duration of the outgrowth time course, with an exponentially increasing amount of virus. These data suggest that our HIV-1 specific RT-PCR requires a level of viral replication achieved after a nearly one week and is not sensitive enough to detect replication defective viruses that do not expand further.

The MOLT4/CCR5 viral outgrowth assay does require a single large blood sample of 150–200 mL. This is due to the low frequency of replication-competent proviruses harbored within resting CD4^+^ T cells. Given this fundamental aspect of the biology of HIV-1 latency, it is unlikely that any version of a viral outgrowth assay can be performed without a large input of resting CD4^+^ T cells. However, because no alternative assay exists that specifically measures latent, replication-competent proviruses in resting CD4^+^ T cells, viral outgrowth assays will likely continue to play an important role in evaluating HIV-1 eradication strategies.

The MOLT-4/CCR5 utilizing viral outgrowth assay presented here is the most rapid and scalable assay available for measuring the size of the HIV-1 latent reservoir. As such, we believe that this assay will be an indispensable tool in evaluating the success of strategies to perturb or eradicate the HIV-1 latent reservoir.

## Materials and Methods

### Patient cohort

Twenty HIV-1 infected patients were enrolled in this study; 19 were recruited from the Moore Clinic at The Johns Hopkins Hospital and 1 patient was recruited from the SCOPE cohort at the University of California San Francisco. All study participants provided written informed consent for participation. This study was approved by the Johns Hopkins Institutional Review Board. Seventeen of the 20 patients were recruited on the basis of prolonged continuous suppression of plasma HIV-1 viremia on HAART to below the limit of detection of standard clinical assays (<50 copies HIV-1 RNA/mL). Three of the 20 patients were recruited on the basis of detectable plasma HIV-1 viremia; two patients were reported non-adherent to their HAART regimens and one patient had not yet initiated therapy.

### Isolation of resting CD4^+^ T lymphocytes

Peripheral blood mononuclear cells (PBMC) were isolated using density gradient centrifugation. CD4^+^ T lymphocytes were enriched by negative depletion (CD4^+^ T cell Isolation Kit, Miltenyi Biotec). Resting CD4^+^ T lymphocytes were further enriched through negative depletion of cells expressing CD69, CD25, or HLA-DR (CD69 MicroBead Kit II, Miltenyi Biotec; CD25 MicroBeads, Miltenyi Biotec; Anti-HLA-DR MicroBeads, Miltenyi Biotec). Resting CD4^+^ T cells purified using the above described two-step bead depletion procedure were stained with CD4-PE and HLA-DR-APC (BD Biosciences). The purity of these cells was analyzed by flow cytometry using a FACS Canto II (BD Biosciences) and FlowJo software (Treestar).

### Limiting dilution viral outgrowth assay

For each patient, both a standard viral outgrowth assay and a MOLT-4/CCR5 viral outgrowth assay were performed using freshly purified resting CD4^+^ T lymphocytes obtained from a single blood draw as described above. Briefly, five-fold serial dilutions of resting CD4^+^ T lymphocytes from HIV-1 infected patients were stimulated by co-culture with a 10-fold excess of γ-irradiated allogeneic PBMC from uninfected donors and the mitogen PHA (Remel) in RPMI containing 10% fetal bovine serum, 100 U/mL IL-2 (Novartis) and 1% T-cell growth factor (produced in house, as described previously [21]). These conditions are sufficient to activate 100% of the resting CD4^+^ T lymphocytes, as previously demonstrated by CFSE dilution and expression of cell surface activation markers [6], [37]. T cell activation reverses HIV-1 latency in at least a fraction of the latently infected cells. After one day of stimulation, the mitogen containing media is removed and either MOLT-4/CCR5 cells or health donor CD4^+^ lymphoblasts are added in fresh media to propagate replication-competent HIV-1 in the culture wells. The standard viral outgrowth assay utilizes two additions of CD4^+^ lymphoblasts from uninfected donors as target cells for HIV-1 outgrowth on days 2 and 9. The MOLT-4/CCR5 viral outgrowth assay utilizes a single addition of MOLT-4/CCR5 cells on day 2. The ratio of target cells added is the same for both assays [21], with 1×10^7^ target cells added to wells containing 1×10^6^ patient resting CD4^+^ T cells and 2.5×10^6^ target cells added to all other wells. Five days after initial mitogen stimulation of input resting CD4^+^ T lymphocytes, the culture media was changed and the cells in each well were split. Supernatants from each well were tested for HIV-1 RNA and/or HIV-1 p24 protein at various time points by RT-PCR and ELISA (Alliance HIV-1 p24 antigen ELISA Kit, Perkin Elmer), respectively. The frequency of latently infected cells among the input resting CD4^+^ T lymphocytes was calculated by a maximum likelihood method, as described previously and is expressed as infectious units per million cells (IUPM) [21]. The 95% confidence intervals for individual IUPM determinations are ±0.7 log IUPM, or 5 fold [21]. With a sample size of 25, this assay can detect with 80% power a 0.2 log reduction in the reservoir assuming a type 1 error of 0.05.

### HIV-1 RNA detection by RT-PCR

After reversal of latency and subsequent release of HIV-1, virion-associated HIV-1 RNA was isolated from 60 µL of culture supernatant using the ZR-96 Viral RNA Kit (Zymo Research), a 96-well column based RNA isolation kit. cDNA was synthesized from the isolated HIV-1 RNA using the SuperScript III First-Strand Synthesis System (Invitrogen) with oligo-dT primers. Isolated cDNA was assayed for HIV-1 by RT-PCR as described previously [26] using an Applied Biosystems 7300 Real Time PCR System (Applied Biosystems) and TaqMan Universal PCR Mastermix (Applied Biosystems). RNA copy number was determined using a standard curve generated with the DNA plasmid pVQA, described previously [26]. RNA copy numbers below 10 copies or above 10^6^ copies were extrapolated based on the standard curve generated.

### Statistics

Infected cells frequencies in limiting dilution assays were calculated as described by Myers et al. [38]. Where appropriate, results were expressed as mean ± standard deviation. A Wilcoxon rank sum test (paired comparison) was used for statistical analysis. All statistical analyses were performed with MedCalc software, v12.4.0.0. A p value of <0.05 was considered significant.

## Supporting Information

Table S1 **IUPM values obtained for the patients enrolled in this study.**
(EPS)

Figure S1 **Purity staining of resting CD4^+^ T cells obtained from an HIV-1 infected patient via two-step bead depletion procedure.** Resting CD4^+^ T cells obtained from an HIV-1 infected patient on suppressive HAART. (**A**) Representative dot plot indicating CD4 and CD25 expression. (**B**) Representative dot plot indicating CD69 and HLA-DR expression.
(EPS)
